# How Precise Is Dental Volumetric Tomography in the Prediction of Bone Density?

**DOI:** 10.1155/2012/348908

**Published:** 2012-05-30

**Authors:** Hakan Bilhan, Selda Arat, Onur Geckili

**Affiliations:** Department of Prosthodontics, Faculty of Dentistry, Istanbul University, 34093 Çapa Istanbul, Turkey

## Abstract

*Objectives*. The aim of this study was to review the bone density assessment techniques and evaluate the macroscopic structure of bone specimens scored by Hounsfield Units (HUs) and decide if they are always in congruence. *Methods*. The mandible of a formalin-fixed human cadaver was scanned by dental volumetric tomography (DVT) for planning of the specimen positions and fabrication of a surgical guide and a surgical stent was fabricated afterwards. Bone cylinders of 3.5 mm diameter and 5 mm length, were excised from the mandible using the surgical stent with a slow speed trephine drill. After removal of the cylinders two more scans were performed and the images of the first scan were used for the determination of the HU values. The removed bone cylinder was inspected macroscopically as well by micro-CT scan. *Results*. The highest HU values were recorded in the interforaminal region, especially in the midline (408–742). Posterior regions showed lower HU values, especially the first molar regions (22–61 for the right; 14–66 for the left first molar regions). *Conclusion*. Within the limitations of this pilot study, it can be concluded that HU values alone could be a misleading diagnostic tool for the determination of bone density.

## 1. Introduction

Dental implants play an important role in the treatment of partial or complete edentulism. Although the success is very high, posterior maxilla can withstand lower mechanical forces because of its thinner cortical layer, and the lower density of the maxillary spongiosa [1] thus is more critical than other sites. Furthermore, the maxillary sinus restrict the available bone volume, necessitating shorter implants and/or grafting procedures [2].

Bone density plays an important role in planning of implant dentistry in terms of timing of loading as well as number of implants to be used for denture support especially in critical locations such as posterior maxillae. Bone mass, structural properties (macro- and microarchitecture), and material properties (modulus of elasticity, mineral density, etc.) constitute mechanical competence of bone, which is commonly referred to as bone quality in implant dentistry [3]. Substantial variations in bone quality in corresponding anatomical sites and direct correlation between bone quality and implant success rates exist [4]. Since mechanical behavior of bone is a critical factor in the attainment and maintenance of osseointegration, several classification systems and procedures were advocated for assessing bone quality and predicting prognosis [5, 6].

These assessment methods have various limitations in addressing cortical and cancellous bones in a subjective and quantitative manner. There are several radiological methods for bone mineral density (BMD) measurements yielding close relationships such as dual-X-ray absorptiometry (DXA) scanning [7] and Hounsfield unit (HUs) from computed tomography imaging [7]. Texture analysis has been applied in micro-CT [8], while Hounsfield units (HU) have been used in spiral CT as a measure, related to jaw BMD [9]. Cone beam computed tomography (CBCT) is a more recent development than spiral CT. Its clinical application in the field of dentomaxillofacial radiology is gaining importance and spreading widely [10–12], but the available research on CBCT-based bone quality assessment is scarce. Low dose CBCT is often advised for implant planning, considering the possibility to gather clinically relevant 3D data at a low dose, but CBCT does not necessarily allow reliable and accurate bone quality assessment when focusing on the inherent radiographic density information that is otherwise expressed by HU [13]. On the other hand, a very recent study reported of a strong positive correlation of radiographic bone density assessed by CBCT with bone volumetric fraction assessed by micro-CT at the site of dental implants in the maxillary bones [14].

Although strong correlations exist, still a 30–50% of unaccounted variance in mechanical properties from bone density measurement has been reported [15, 16]. Osteoporotic cancellous bone is characterized by low bone mass as well as a deterioration of the microarchitecture. Whether a patient is diagnosed osteoporotic depends only on his/her BMD and how this BMD value compares to a population average [17]. Clinical results have shown that BMD of patients with osteoporotic bone fractures and patients without such fractures can have a substantial overlap [18], causing variance similar to that in density—mechanical property relationship studies. The microarchitecture of cancellous bone has been largely attributed to this variance; density approximates the amount of bone tissue within a cancellous bone specimen but it does not quantify the microarchitecture that is inherent. Together with bone density readings, a quantitative measurement of microarchitectural parameters may improve our ability to better estimate bone strength [19]. Microcomputed tomography (micro-CT) is a relatively new method to image and quantify bone with very high resolution [14, 20]. Microcomputed tomography (micro-CT) scanners, with similar working principle as conventional clinical CT scanners, have been used to study microstructure of materials in three dimensions and to study the relationship between microarchitectural parameters [21] and mechanical properties of cancellous bone [22]. Micro-CT may not be applicable routinely in clinical practice for now, although as a reliable method for bone mass and structure evaluation, it might offer much needed insight into bone quality assessment by providing objective and quantitative microstructural data. Image datasets of human cancellous bone specimens at micron resolution were acquired and from these datasets, microarchitectural parameters have been determined and converted into microfinite element (FE) models [23]. It was shown that the predictive power of bone strength and stiffness was improved with the combination of bone density and microarchitecture information and this work supported the prediction of microarchitecture using current clinical computed tomography imaging technology [23].

While clinical CT scanners typically produce images composed of 1 mm^3^ volume elements (voxels), X-ray microcomputed tomography (micro-CT or *μ*CT) systems developed in the early 1980s had much better spatial resolution, producing voxels in the range of 5–50 *μ*m, or approximately 1,000,000 times smaller in volume than CT voxels [24, 25]. Early micro-CT scanners were custom-built and not widely available. Compact commercial systems are now available and are rapidly becoming essential components of many academic and industrial research laboratories. A wide range of specimens may be examined directly using Micro-CT including mineralized tissues such as teeth, bone, and materials such as ceramics, polymers, or biomaterial scaffolds [26].

Micro-CT systems are now widely used in many academic fields, several recent reviews have presented the current state of micro-CT imaging and analysis of them [14, 27–30].

Emphasis has traditionally been placed on the cortical bone as quality predictor due to its stiffness for achieving primary stabilization [31, 32]. However, a dental implant is mainly in contact with cancellous part of bone, and mechanical characteristics of cancellous bone also influence the load bearing capacity of implant-bone union. In fact, the presurgical determination of bone density plays an important role in planning of the surgical procedure as well as prosthetic treatment. In another study human cadaveric maxillary and mandibular trabecular bone with 3D morphometric data acquired through micro-CT were analyzed and correlated with bone density measurements in Hounsfield scale and Lekholm-Zarb bone classification [3, 5].

Micro-CT is a nondestructive, fast, and precise technique that allows measurements of trabecular and cortical bone [26]. It can provide a spatial representation of bone formation at the implant surface and the peri-implant region up to a few microns or even better, and can evaluate both qualitative and quantitative morphometry of bone integration about dental implants [33].

The aim of this pilot study was to review the current literature about bone density assessment and evaluate the macroscopic structure of bone specimens having been scored by Hounsfield units afore.

## 2. Material and Methods

### 2.1. Experimental Protocol

#### 2.1.1. Preoperative CBCT Imaging

**Figure 1:**
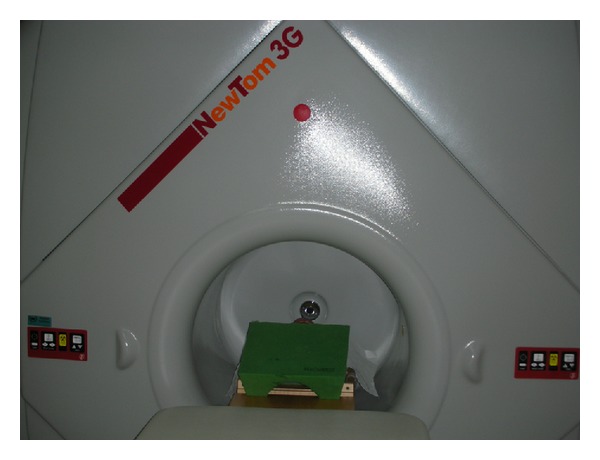
Newtom cone beam 3D imaging equipment.

**Figure 2:**
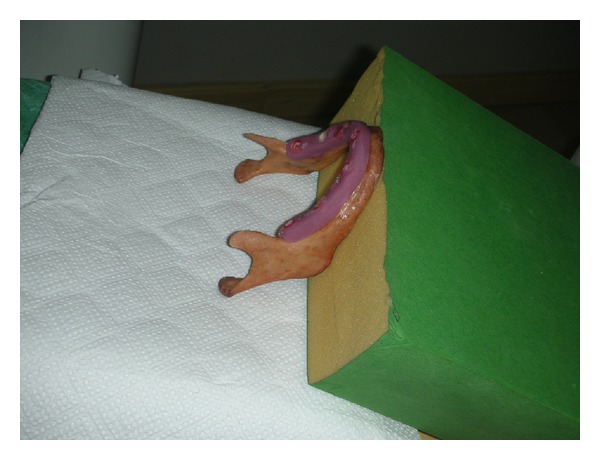
The surgical guide which was fabricated on the cast obtained from an impression of the alveolar process of the mandible.

The mandible of a formalin-fixed human cadaver was involved in this study. After removal of the mandible from the cadaver, the obtained mandible was scanned by dental volumetric tomography (DVT) for planning of the specimen positions and fabrication of a surgical guide. The scan of the mandible was performed with a Newtom (Newtom Cone Beam 3D Imaging, AFP Imaging Corporation, New York, USA; Figure 1). A surgical guide was fabricated on the cast obtained from an impression of the alveolar process of the mandible (Figure 2).

#### 2.1.2. Specimen Preparation

**Figure 3:**
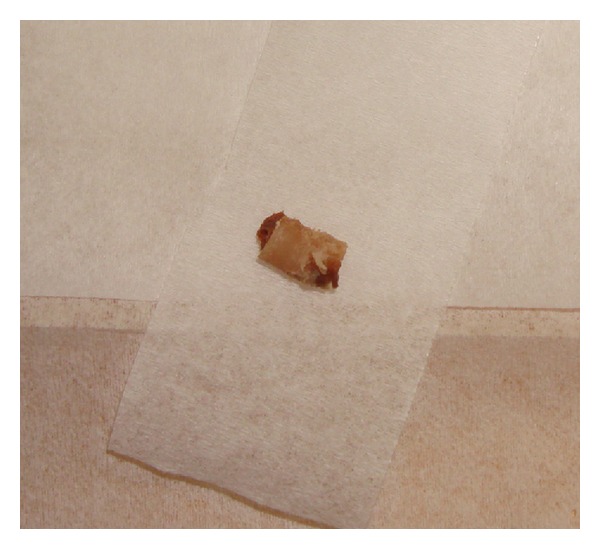
Excised bone cylinders using the surgical stent.

**Figure 4:**
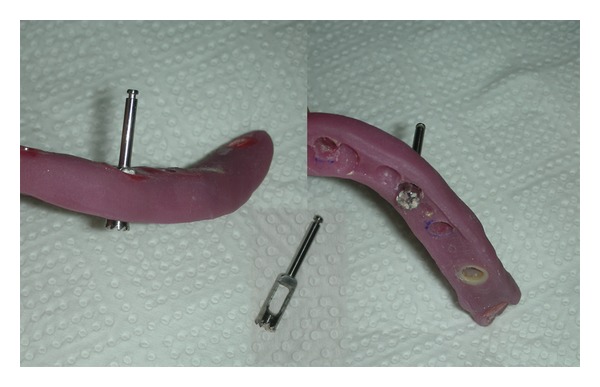
Excision of bone cylinders from the mandibular body using a slow speed trephine drill.

Bone cylinders of 3.5 mm diameter and 5 mm length, were excised from a cadaver mandible using the surgical stent (Figure 3). Excising of bone cylinders from the mandibular body was done using a slow speed trephine drill (Trephine Drill 3.5 mm × 22 mm, Salvin Dental Specialties, Inc, Charlotte, NC, USA) under constant irrigation by the use of the prepared stent (Figure 4). Nine bone cylinders in total were extracted for the experiment; cylinders were stored in consequently numerated 0.9% saline solution containing little vessels. However, since 2 of the cylinders were damaged during removal, these were excluded from the study. The 5 mm cylinder length was chosen to satisfy continuum assumption, so that mechanical properties derived for each cylinder was representative of the whole bone [34].

#### 2.1.3. Postoperative CBCT Imaging and Determination of the Hounsfield Units

After removal of the cylinders two more scans were performed, with the surgical stent and without surgical the stent, respectively. The images of the first scan were used for the determination of the Hounsfield Unit values. The cavities of the removed bone specimens were localized by viewing the second and third scan images and the Hounsfield Unit values of the missing bone cylinders was determined by taking the mean of five values: coronal, apical, buccal, lingual, and the center. The software (Newtom Imaging Software, AFP Imaging Corporation, New York, USA) is capable of giving graphically the HU values of the marked zone. For each bone cylinder, the raw CT values were converted into HU by means of the following formula [35]: HU = 1000(CT − CT_*w*_)/(CT_*w*_ − CT_*a*_) where CT, CT_*w*_, and CT_*a*_ are the values of bone, saline, and air, respectively.

#### 2.1.4. Micro-CT Imaging

**Figure 5:**
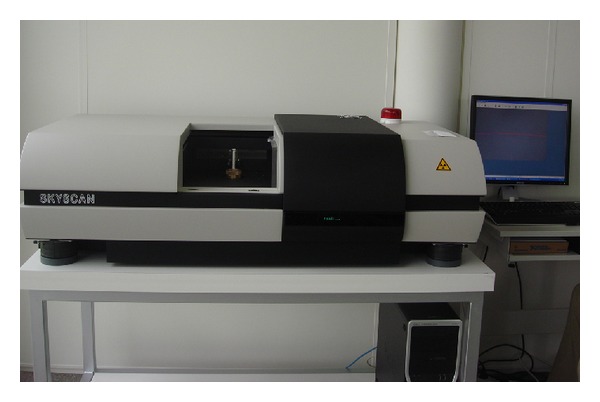
The photograph of the micro-CT scanner.

**Figure 6:**
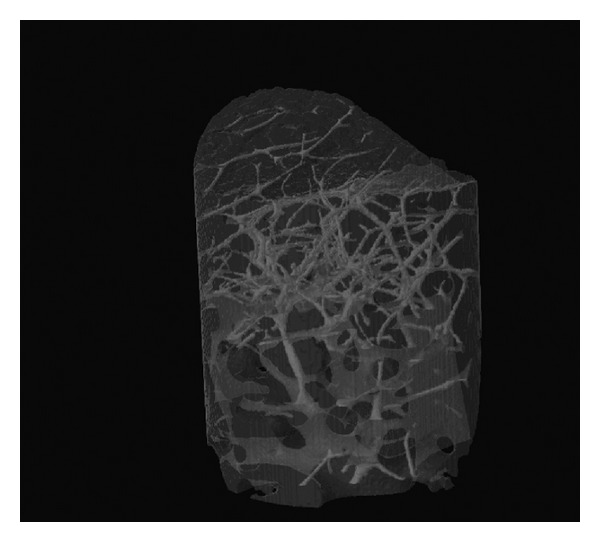
The micro-CT image of the cylinder-shaped bone specimen.

As a part of the pilot study, the bone cylinder on the left first premolar molar region of human cadaver bone was randomly chosen for micro-CT scanning. The bone cylinder was placed in a custom vessel wrapped in paper soaked with saline solution to prevent any desiccation, and isotropically scanned at 14 *μ*m resolution with a model 1172 micro-CT scanner (Skyscan, Kontich, Belgium) using a CBCT scanning technique (Figure 5). CBCT is a novel CT image acquisition technique in which up to a several hundred CT images (as opposed to 1–3 images in normal CT) are reconstructed by one data acquisition as the data on the fluoroscopic image is handled as plane data. The total time required for scanning and reconstruction was approximately 30 minutes per sample, thus deterioration of the bone cylinder as a result of being exposed to ambient conditions was significantly reduced. To ensure a consistent CT image resolution among all the datasets, the scanner turntable location was fixed at a specific SOD and SID distance of 19.03 mm and 356.90 mm, respectively. The X-ray parameters were set at 51 kV and 200 *μ*A and the CT images were processed at a scaling coefficient of 50 and averaged three times. With these parameters, together with a 0.5 mm aluminum plate placed at the X-ray detector, a good contrast was achieved in the resultant CT images between trabeculae. Resultant dataset had an isotropic resolution of 14.836 *μ*m. Resultant CT images for each bone cylinder was evaluated for microarchitectural parameters [19] such as tissue volume, bone volume, percent bone volume, tissue surface, bone surface, intersection surface, bone specific surface, bone surface density, trabecular bone pattern factor, structure model index, trabecular thickness, trabecular number and trabecular separation (CT Analyzer, Skyscan, Belgium) (Figure 6).

## 3. Results

### 3.1. Relationship between Microarchitectural Parameters and Hounsfield Unit and Interrelationship between Microarchitectural Parameters

The main objective of this study was to study the possibility of inferring of microarchitectural parameters from clinical CT images.

The rate of cancellous bone volume in the total volume of core is the bone volume density (BV/TV), which was in the range of 0.12–0.29 for the sample.

All the measured HU values, means, and ranges are shown in Table 1. The highest HU values were encountered in the interforaminal region, especially in the midline (408–742). Posterior regions showed lower HU values, especially the first molar regions (22–61 for the right; 14–66 for the left first molar regions). The bone cylinder which was scanned by micro-CT showed an incongruous structure when HU of the donor site was considered. The HU values indicated a higher density bone, whereas the Micro CT image revealed rather a spongious bone (Figure 6).

## 4. Discussion 

Our primary objective of studying the relationship between HU from CT images and microarchitectural parameters, density given in objective values (mg/cm^3^) and HU, was the evaluation of a diagnostic tool for in vivo assessment of bone quality. 

A formalin-fixed cadaver mandible was harvested and used in the present study for several reasons. The main reason was the ease to obtain in comparison to a fresh cadaver as recommended in a few studies [36, 37]. Another reason was the protection from communicable diseases. Tissue fixation with 10% formalin (4% formaldehyde) is widely used to preserve specimens without refrigeration, offering researchers the added benefit of protection from specimens with communicable diseases [38–42]. Although it is assumed that formalin fixation alters the mechanical properties of bone, studies failed to deduce quantitative data [43, 44]. Chemical fixation through the use of aldehydes has been shown to cause a direct effect on bone mechanical properties by forming an increased number of inter- and intrafibrillar cross-links of primary amine groups of polypeptide collagen chains [38, 44] have shown that while formalin fixation has no effect on the mineral composition of bone, it causes the collagen fibrils to be more closely packed. However, in a recent study it was reported that formalin fixation and freezing would not adversely affect the viscoelastic and elastic mechanical properties of murine bone [45]. The use of embalmed bone is known to be used in studies testing the mechanical behavior and efficacy of fracture fixation devices, joint prostheses, and other reconstructive orthopedic devices [46].

Accuracy of micro-CT was qualitatively evaluated by comparing to standard histomorphometric data with the corresponding CT slices for the same specimen. The results showed that, in general there was a good correlation between histomorphometric data and microtomographic data. One author obtained a correlation coefficient of 0.855 [33]. 

The result from this pilot study has raised doubt that in addition to bone density, bone microarchitectural parameters can also be predicted from clinical-CT imaging. We chose to use the CBCT, as it is a three-dimensional measurement often used in dentistry. In similar studies [47, 48], it was reported that to reasonably evaluate cancellous bone architecture, image datasets of resolutions not more than 100 *μ*m should be used. Microarchitectural parameters from both clinical-CT imaging and histological sections were also compared and it was shown that high-resolution clinical-CT resulted in an overestimation of microarchitectural parameters. In a recent study [3], the scan was initially utilized to assess bone quality subjectively in Lekholm and Zarb classification [5] at incisor and molar edentulous sites by rating the distribution of cortical and cancellous bones and density of cancellous bone in HU was determined through a function of the CT equipment by averaging the readings of multiple slices within respective sites. Similarly, in our study, the Hounsfield units were determined by taking the average of five values of the removed cylinders: coronal, apical, buccal, center, and lingual, additionally stating the range of HU values of each cylinder. 

For harvesting the cylinders a trephine drill was used as described in another study [3]. There is great difficulty in accurately excising bone specimens that correspond to the exact CT volume of interest, if the bone specimens are to be excised after clinical imaging, as pointed out in a previous study [23]. In the previously mentioned study, the specimens were scanned after removal from the bone for this reason. However in the present study we preferred to use the whole mandible in the CBCT to mimic the clinical application. 

In a study by Fanuscu and Chang [3], the anterior sites in both arches were noted to be volumetrically denser than the posterior sites, indicating varying bone mass. It was noted that volume density remained depthwise stable in the maxilla, whereas in the mandible it decreased with depth in the corono-apical direction, as being seen in our study too. 

Current classifications and procedures for evaluating bone have certain shortcomings as mechanical competence in terms of mass, structure and material is not well addressed for trabecular bone. There have been unsuccessful attempts to quantify bone density in consideration of mechanical strength. Friberg et al. [49] proposed an objective cutting resistance procedure that might provide a composite value for mechanical characteristics in predicting bone quality for initial stability. However, mechanics of drilling with a bur and withstanding occlusal forces by an implant has to be further investigated and correlated. Trisi and Rao [50] compared histomorphometrics and hand-felt cutting resistance and demonstrated that subjective tactile sensation was proved to be poor in discerning finer differences. 

About ten years ago an image-based bone density classification that utilizes gray-scale values through CT was suggested [51]. The method of preoperative bone density measurement was advocated as a prognostic indicator in which site-specific, objective and quantitative results on the Hounsfield scale would provide bone-quality information. Following this perspective, the reliability of Hounsfield units in predicting bone density was evaluated in this preliminary study. The proposed classification evaluates bone mass; however, its mechanical value is limited without structural and material properties. Riggs et al. [52] reported on bone mass increase in osteoporotic patients by medications and found that bone strength was not increased and fracture risk was not lowered as much as expected by the gain in bone density. This suggests that there is an important influence of the complex microarchitecture on the mechanical competence of bone. 

It should be underlined that CBCT data have a larger amount of scattered X-rays than conventional spiral CT. This may enhance the noise in reconstructed images, and thus affect the low contrast detectability [53]. Because of scatter and artifacts, HU values in CBCT are not valid, and therefore the method of correlating BMD to HU values from CBCT is not ideal. Moreover, the scatter and artifacts in CBCT get worse around inhomogeneous tissues with reduced HU values up to 200 HU [54], which confirms that the HU in CBCT is not a valid method for bone quality assessment. Since up till now CBCT-based bone quality assessment is neither accurate nor reliable, there is a need to find methods to circumvent the shortcomings of this particular development, so as to have a reliable way to assess bone quality or there is a need for methods, other than density measurements, for bone quality assessment. In a bone density assessment study it was concluded that mandibular cortical bone was denser than cortical bone of the upper maxilla, whereas cancellous bone has similar densities in both mandible and the upper maxilla. The main problem appears to lie in the differentiation of tissues of similar density [14]. Texture analysis may thus come into play, which is strengthened by the fact that bone quality may be expressed by its microarchitectural composition. May be for this reason a very recent study concluded that correlation of micro-CT and conventional histomorphometry should be subject of future research [14]. In contrast to classic histomorphometry architectural metric parameters such as bone volume (BV), total volume (TV), and bone surface can be directly determined from the 3D images acquired by micro-CTs, without assuming the geometric model [55]. 

The increased failure rate of implants that are placed in posterior regions was attributed to differences in bone quality and quantity and elevated occlusal stress in the molar areas [56, 57]. Therefore it is very important to analyze the bone density in posterior maxillae before implant surgery. 

Within the limitations of this pilot study, it can be concluded that HU values alone could be a misleading diagnostic tool for the determination of bone density. It is advisable to concentrate future research on density quantification from clinical CT images and relate those to various bone types with different mechanical properties to be able to make predictions concerning the bone quality. 

## Figures and Tables

**Table 1:** HU values of the specified regions.

Region	HU values					HU range	HU mean
1	84	40	50	74	66	40–84	62,8
2	32	61	44	30	22	22–61	37,8
3	164	92	74	129	155	74–164	122,8
4	234	285	378	354	308	234–378	311,8
5	742	614	534	586	408	408–742	576,8
6	384	425	331	285	456	285–456	376,2
7	124	185	98	82	155	82–185	128,8
8	24	66	62	14	26	14–66	38,4
9	84	107	42	34	26	26–107	58,6

Region 1: right retromolar pad region of human cadaver bone.

Region 2: right first molar region of human cadaver bone.

Region 3: right first premolar molar region of human cadaver bone.

Region 4: right lateral region of human cadaver bone.

Region 5: midline (symphysis).

Region 6: left lateral region of human cadaver bone.

Region 7: left first premolar molar region of human cadaver bone.

Region 8: left first molar region of human cadaver bone.

Region 9: left retromolar pad region of human cadaver bone.
